# Aortic stiffness and cardiac function in middle-aged women with ischemic stroke with and without migraine

**DOI:** 10.1186/s12883-026-04960-z

**Published:** 2026-05-23

**Authors:** Mariam Ali, Ghislaine Holswilder, Nelleke van der Weerd, Hendrikus J.A. van Os, Katie M. Linstra, Thijs W. van Harten, Bastiaan J.C. te Kiefte, Sophie D.M. Laveaux, Birgitta K. Velthuis, Nyika D. Kruyt, Jos J. M. Westenberg, Antoinette MaassenVanDenBrink, Gisela M. Terwindt, Hildo J. Lamb, Marieke J.H. Wermer

**Affiliations:** 1 Department of Neurology, Leiden University Medical Centre, P.O. Box 9600, Albinusdreef 2, Leiden, 2300 RC the Netherlands; 2 Department of Radiology, Leiden University Medical Center, Leiden, the Netherlands; 3 Department of Human Genetics, Leiden University Medical Centre, Leiden, the Netherlands; 4 Department of Public Health & Primary Care, Leiden University Medical Centre, Leiden, the Netherlands; 5 Department of Neurology, Massachusetts General Hospital, Harvard Medical School, Boston, USA; 6 Department of Radiology, University Medical Center Utrecht, Utrecht, the Netherlands; 7 Department of Internal Medicine, Erasmus MC University Medical Centre, Rotterdam, the Netherlands; 8 Department of Neurology, University Medical Centre Groningen, Groningen, the Netherlands

**Keywords:** Migraine, Ischemic stroke, Women, Macrovascular Status, Cardiac Function, Aortic Stiffness

## Abstract

**Introduction:**

Migraine increases the risk of ischemic stroke and cardiovascular disease in women, potentially due to systemic vascular dysfunction. We investigated whether migraine is associated with increased aortic stiffness and impaired cardiac function as markers of macrovascular dysfunction in middle-aged women with ischemic stroke.

**Methods:**

This cross-sectional study included three groups of women aged 40–60 years with: (I) ischemic stroke, (II) ischemic stroke with migraine, and (III) no history of stroke or migraine. Aortic stiffness was measured by assessing aortic arch pulse wave velocity (arch PWV) using high-temporal 2D-phase contrast MRI. Cardiac function was evaluated through left ventricular measures, including stroke volume (SV), cardiac output (CO), and ejection fraction (EF), from two orthogonal cine biplane long-axis (2-/4-Chamber) acquisitions. Multivariable linear regression was used to estimate mean differences in arch PWV and left ventricular function, comparing groups 1 and 2 with group 3 as the reference.

**Results:**

Among 118 included women (mean age 51 years [SD 5]), (I) 44 had ischemic stroke, (II) 44 had both ischemic stroke and migraine, and (III) 30 had no stroke or migraine. In the reference group (group III), mean (SD) values were 6.97 (1.05) m/s for arch PWV, 95.91 (15.82) mL for SV, 6.73 (1.61) L/min for CO, and 64.90 (5.96)% for EF. Comparisons of groups I and II with group III showed no statistically significant mean differences in arch PWV, SV, CO, or EF. Although mean arch PWV was slightly higher in group II (7.11 [1.53] m/s), the adjusted mean difference (-0.03 m/s; 95%CI: -0.60 to 0.54 m/s) was not statistically significant. Small, non-significant reductions in SV, CO, and EF were also observed in the ischemic stroke groups compared with the reference group.

**Conclusions:**

The increased risk of ischemic stroke in middle-aged women with migraine was not associated with differences in arch PWV or left ventricular function in this cohort. We did not detect evidence that macrovascular dysfunction plays a key role in linking migraine and cardiovascular risk, supporting other studies that point to a potential role for microvascular dysfunction.

**Supplementary Information:**

The online version contains supplementary material available at 10.1186/s12883-026-04960-z.

## Background

Migraine is three times more prevalent in women than in men and is a well-established risk factor for ischemic stroke, especially in those with aura [1–3]. In men, however, this risk is less certain [1–3]. Migraine is also associated with an increased risk of systemic cardiovascular diseases, including myocardial infarction and peripheral arterial disease [4]. This risk is particularly pronounced in young women and is not explained by large-artery atherosclerosis, traditional cardiovascular risk factors, or hormonal contraceptive use [5–8]. The mechanisms underlying this association are unclear and probably multifactorial. One potential mechanism is a systemic vascular dysfunction—possibly influenced by female sex hormones—which may act through both macrovascular and microvascular pathways [9]. While previous studies suggest an association between migraine and microvascular pathology, the potential link between migraine and macrovascular pathology remains unexplored [9]. 

Non-invasive markers of macrovascular health, such as aortic arch pulse wave velocity (arch PWV), are more often elevated in migraine patients and reflect large artery (aortic) stiffness [10–12]. We selected arch PWV as the primary marker of macrovascular pathology because it is a well-established, noninvasive measure of aortic stiffness and provides direct information on large-artery properties that may be relevant to cerebral hemodynamics and ischemic stroke risk. Aortic stiffness increases cardiovascular risk by adding cardiac workload and afterload, which can elevate blood pressure and contribute to ischemic stroke risk [13]. We included LV systolic function as a secondary measure because it reflects the functional hemodynamic consequences of increased arterial stiffness through ventricular–arterial interaction, rather than suggesting a direct myocardial association with migraine. Impaired cardiac function, particularly reduced left ventricular (LV) systolic function, can also reflect macrovascular involvement [13]. While aortic stiffness and LV systolic function are separate markers of macrovascular health, increased afterload caused by stiff arteries can impair LV function by reducing the heart’s ability to pump blood efficiently. This in turn can increase ischemic stroke risk through diminished blood flow and potential clot formation [13]. Although a direct myocardial involvement in migraine has not been established, migraine-related macrovascular alterations, such as increased arterial stiffness, may be reflected in subtle changes in LV systolic performance due to changes in arterial afterload. Together, arch PWV and LV systolic function provide complementary information, with arch PWV reflecting the vascular component of macrovascular dysfunction and LV systolic function reflecting its functional cardiac consequences. Both arch PWV and LV function can be assessed through magnetic resonance imaging (MRI), a technique that does not involve ionizing radiation, allowing standardized and noninvasive evaluation of both vascular and cardiac measures within a single examination.

In this study, we first investigated whether middle-aged women with both ischemic stroke and migraine or ischemic stroke alone have increased aortic stiffness, compared with women without stroke or migraine. Impaired cardiac function was assessed as a secondary outcome to reflect the hemodynamic consequences of arterial stiffness.

## Methods

### Study design and participants

This study included women aged 40–60 from two cross-sectional studies conducted at the Leiden University Medical Center (LUMC) between October 2016 and December 2021, using identical study protocols: (I) the Cardiovascular RiskprofilE in Women – MIcrovascular STatus (CREW-MIST) and (II) the WHIte matter lesions in young to middle-aged women with Stroke, PreEclampsia, and migRaine (WHISPER) [14, 15]. The age range of 40–60 was selected to ensure a relatively homogeneous population at risk for stroke, while minimizing the confounding effects of age-related cardiovascular risk factors and atherosclerosis. Participants were categorized into three groups: (I) women with a history of ischemic stroke, (II) women with a history of both ischemic stroke and migraine, and (III) women without a history of ischemic stroke or migraine.

Groups I and II were derived from the CREW-MIST study, which recruited participants meeting the inclusion criteria from neurology clinics of the LUMC and University Medical Center Utrecht, as part of the CREW Consortium. Data collection and MRI took place at LUMC. Ischemic stroke was defined as a sudden episode of focal neurological deficits lasting more than 24 h, with corresponding lesions on CT and/or MRI, based on medical records, discharge summaries, and neuroimaging reports, and occurring at least three months prior to inclusion. Stroke subtypes were classified according to the Trial of ORG 10,172 in Acute Stroke Treatment (TOAST) criteria, based on the same sources [16]. Stroke location details were extracted from medical records. Both groups were screened for migraine using the International Classification of Headache Disorders-III (ICHD-III) criteria [17]. Women in group II were diagnosed with lifetime migraine, either with or without aura, according to these criteria. In line with our previous CREW-MIST and WHISPER substudy, migraine status was determined through semi-structured interviews conducted by trained researchers and classified according to the ICHD-III criteria [14]. In cases of doubt, a specialized headache neurologist (G.M.T.) reviewed the clinical information and made a final diagnosis.

Group III served as a reference group and was derived from the WHISPER study conducted at the LUMC, which included participants without a history of ischemic stroke or migraine, through advertisements by the Dutch Heart Foundation. All groups excluded women with other neurological disorders, systemic diseases affecting white matter, demyelinating or inflammatory conditions, retinal or cerebral venous infarctions, pre-eclampsia, polycystic ovary syndrome, pregnancy, illnesses affecting participation, contraindications for MRI, or insufficient proficiency in Dutch or English.

As part of the studies, participants completed questionnaires on demographics, medication use and medical history and underwent a 3T MRI scan and blood collection during a one-day research visit. For participants with migraine, the visit was postponed if a migraine attack occurred on that day.

### Cardiovascular magnetic resonance (CMR) imaging

#### Image acquisition

CMR imaging was conducted on a Philips 3T MRI scanner, using a body coil for transmission and a 28-element phased array surface coil on the chest for signal reception. The imaging protocol included measurements of arch PWV using high-temporal 2D phase contrast MRI and LV systolic function through orthogonal cine biplane long-axis acquisitions (2- and 4-chamber views). Long-axis cines were acquired during breath-holds using electrocardiogram-gated balanced steady-state free precession sequences. Arch PWV analysis was performed on velocity-encoded CMR scans of the ascending and descending aorta. Imaging parameters were as follows: echo time/repetition time 1.44/2.88 ms, flip angle 45°, slice thickness 8 mm, one slice, 40 phases, 450 × 400 mm² field-of-view, 230 × 200 mm² matrix size, resulting in a resolution of 336 × 336 pixels with a pixel spacing of 1.18 × 1.18 mm². llustrative cardiovascular MRI images of aortic arch PWV and LV systolic function assessment have been published previously by our group [18–20]. 

### Image analysis

Arch PWV was calculated as the aortic arch length divided by the transit time of the systolic wave arrival, as previously described [21]. Measurements were performed semi-automatically using custom-made MASSx software (version 7.3, Medis Medical Imaging Systems, Leiden, the Netherlands) by two independent observers (G.H. and S.D.M.L.) supervised by a senior researcher (J.J.M.W., > 20 years of experience in cardiac MRI). The velocity encoding (VENC) setting of the phase contrast MRI was 180 cm/s.

LV systolic function was assessed in QMass software (version 8.1, Medis Medical Imaging Systems). One observer (M.A.) manually outlined LV contours in 2- and 4-chamber views at end-diastole and end-systole, excluding papillary muscles, to calculate stroke volume (SV), ejection fraction (EF), and cardiac output (CO). For inter-observer variability, 10% of the sample was re-evaluated by a second observer (B.J.C.t.K) supervised by J.J.M.W. Observers were blinded to stroke and migraine diagnoses.

### Sample size calculation

The sample size was calculated prior to participant inclusion, based on findings from a previous study that reported a mean arch PWV of 6.5 m/s with a standard deviation of 3.1 m/s in women aged 40–49 years [22]. To detect a clinically relevant absolute difference of 2 m/s in arch PWV between groups, a total of 114 participants (*n* = 38 per group) was needed to achieve 80% power at a two-sided significance level of 5%.

### Statistical analysis

We performed descriptive statistics for baseline characteristics and visually inspected the distributions of arch PWV as the primary outcome and LV systolic function measures (SV, CO, and EF) as secondary outcomes for normality. We conducted linear regression analyses adjusting for age, menopause, smoking (ever), hypertension, hypercholesterolemia, and body mass index (BMI), using complete case analyses. Regression coefficients (β) with corresponding 95% confidence intervals (CIs) were calculated to estimate adjusted mean differences between groups I (ischemic stroke) and II (ischemic stroke with migraine), using group III (no stroke or migraine) as the reference.

### Ethical statement

The Medical Ethics Committee Leiden-Den Haag-Delft approved the study protocols for CREW-MIST (P15.384) and WHISPER (P18.130). Written informed consent was obtained from all participants. 

### Data availability and reporting standards

The data supporting the findings of this study are available from the corresponding author upon reasonable request. We followed the STROBE Statement reporting guideline (see the Data Supplement for the checklist).

## Results

In total, 118 women with complete arch PWV and LV systolic function data were included in this study: 44 (37%) had a history of ischemic stroke (group I, mean age 52 years [SD 5]), 44 (37%) had a history of both ischemic stroke and migraine (group II, mean age 52 years [SD 5], with 19 [43% of group II] having migraine with aura), and 30 (25%) had no history of ischemic stroke or migraine (group III, mean age 51 years [SD 5]; Table 1).

**Table 1:** Baseline characteristics of participants

	I. ischemic stroke only (*n* = 44)	II. Ischemic stroke with migraine (*n* = 44)	III. No ischemic stroke or migraine (*n* = 30)
Age at visit, years (mean (SD))	52.3 (5.1)	52.5 (4.8)	51.4 (4.7)
Age at stroke, years (mean (SD))	46.6 (6.3)	47.2 (5.4)	n.a.
Time between stroke and research visit, years (mean (SD))	5.7 (5.5)	5.3 (5.1)	n.a.
History of intracerebral hemorrhage or TIA	1 / 44 (2.3)	1 / 44 (2.3)	n.a.
White ethnicity	41 / 42 (97.6)	38 / 42 (90.5)	26 / 27 (96.3)
Body mass index, kg/m^2^ (median (IQR))*	27 (24–31)	27 (24–30)	25 (23–28)
Cardiovascular risk factors			
Hypertension	22 / 44 (50.0)	28 / 44 (63.6)	6 / 29 (20.7)
Diabetes mellitus	3 / 44 (6.8)	1 / 44 (2.3)	0 / 29 (0)
Hypercholesterolemia	21 / 44 (47.7)	17 / 44 (38.6)	2 / 29 (6.9)
Smoking	33 / 44 (75.0)	17 / 44 (38.6)	12 / 29 (41.4)
Alcohol use	29 / 44 (69.0)	28 / 42 (66.7)	21 / 27 (77.8)
Medication (ever use)			
Statins	19 / 44 (43.2)	14 / 44 (31.8)	1 / 29 (3.4)
Antihypertensive medication	19 / 44 (43.2)	19 / 44 (43.2)	3 / 29 (10.3)
Platelet aggregation inhibitor	30 / 43 (69.8)	38 / 44 (86.4)	n.a.
Vitamin K antagonist	2 / 43 (4.7)	3 / 44 (6.8)	n.a.
Anticoagulants	35 / 44 (79.5)	40 / 44 (90.9)	n.a.
Gynecological parameters			
Postmenopausal	22 / 44 (50.0)	21 / 44 (47.7)	15 / 29 (51.7)
Age at last menstruation (median (IQR))†	49 (46–52)	49 (46–51)	52 (49–53)
Ever use of combined or progestin-only oral contraceptives	1 / 44 (2.3)	1 / 44 (2.3)	2 / 30 (6.7)
Migraine characteristics			
Migraine with aura	n.a.	19 / 44 (43.2)	n.a.
Migraine without aura	n.a.	25 / 44 (56.8)	n.a.
Age at migraine onset, years (mean (SD))	n.a.	19 (5.3)	n.a.
Triptan use	n.a.	11 / 44 (25.0)	n.a.
Average lifetime migraine attack frequency p/year			
*1–12* (i.e., an average of 1 attack p/month)	n.a.	28 (63.6)	n.a.
*13–54* (i.e., an average of 1–4 attacks p/month)	n.a.	15 (34.1)	n.a.
*> 54* (i.e., several attacks p/week)	n.a.	1 (2.3)	n.a.
Stroke cause according to the TOAST criteria			
Large artery atherosclerosis	12 / 44 (27.3)	9 / 44 (19.6)	n.a.
Cardioembolic	2 / 44 (4.5)	4 / 44 (8.7)	n.a.
Small vessel occlusion	10 / 44 (22.7)	9 / 44 (19.6)	n.a.
Other cause	8 / 44 (18.2)	2 / 44 (4.3)	n.a.
Undetermined cause	12 / 44 (27.3)	20 / 44 (45.5)	n.a.

Data are n / N (%), unless otherwise specified

*Abbreviations IQR* interquartile range, *na* not applicable, *SD* standard deviation, *TIA* transient ischemic attack, *TOAST* Trial of ORG 10,172 in Acute Stroke Treatment,  *y *years

Missing values, n (%): *2 (4.5%) vs. 2 (4.5%) vs. 3 (10%), and †20 (45%) vs. 20 (45%) vs. 13 (43%)

Among stroke patients, those with migraine (group II) were less often smokers and more frequently had hypertension compared with those without migraine (group I). Other baseline characteristics and traditional cardiovascular risk factors were largely similar between the two ischemic stroke groups. However, stroke etiologies differed substantially between groups I and II (Table 1).

### Arch PWV and LV systolic function measures

Mean values of arch PWV, SV, CO, and EF were similar across groups (Table 2). Although the mean arch PWV was slightly higher in group II (ischemic stroke with migraine) at 7.11 (SD 1.53) m/s, the adjusted mean difference (-0.03 m/s) compared with the reference group was not statistically significant (95% CI: -0.60 to 0.54 m/s). Similarly, small reductions in SV, CO, and EF were observed in both ischemic stroke groups compared with the reference group, but none of these differences were statistically significant (Table 2).

**Table 2:** Crude and adjusted mean differences in arch PWV and LV systolic function measures (95% CIs) using women with no history of ischemic stroke or migraine (group III) as the reference

	Mean (SD) levels	Unadjusted β (mean difference with 95% CI)	Adjusted β (mean difference with 95% CI)*
Aortic stiffness			
Arch PWV (m/s)			
1. Ischemic stroke only (*n* = 44)	6.78 (1.46)	−0.15 (− 0.70 to 0.40)	−0.12 (− 0.45 to 0.55)
2. Ischemic stroke and migraine (*n* = 44)	7.11 (1.53)	0.10 (− 0.48 to 0.68)	−0.03 (− 0.60 to 0.54)
3. No ischemic stroke or migraine (*n* = 30)	6.97 (1.05)	Reference	Reference
LV systolic function measures			
Stroke volume (mL)			
1. Ischemic stroke only (*n* = 44)	89.35 (18.94)	−5.80 (− 13.90 to 2.30)	−1.90 (− 8.80 to 5.00)
2. Ischemic stroke and migraine (*n* = 44)	87.36 (20.74)	−7.50 (− 15.60 to 0.60)	−4.90 (− 11.40 to 1.60)
3. No ischemic stroke or migraine (*n* = 30)	95.91 (15.82)	Reference	Reference
Cardiac output (L/min)			
1. Ischemic stroke only (*n* = 44)	5.86 (1.26)	−0.80 (− 1.45 to − 0.05)	−0.20 (− 0.60 to 0.20)
2. Ischemic stroke and migraine (*n* = 44)	5.84 (1.41)	−0.85 (− 1.50 to − 0.20)	−0.10 (− 0.55 to 0.35)
3. No ischemic stroke or migraine (*n* = 30)	6.73 (1.61)	Reference	Reference
Ejection fraction (%)			
1. Ischemic stroke only (*n* = 44)	62.97 (7.17)	−1.70 (− 4.60 to 1.20)	−1.10 (− 3.90 to 1.70)
2. Ischemic stroke and migraine (*n* = 44)	63.34 (7.93)	−1.30 (− 4.20 to 1.60)	−0.90 (− 3.20 to 1.40)
3. No ischemic stroke or migraine (*n* = 30)	64.90 (5.96)	Reference	Reference

*Abbreviations* *CI* confidence interval, *CO* cardiac output,  *EF* ejection fraction, *LV* left ventricular, *m/s* meters per second,  *mL* millimeters,  *PWV* pulse wave velocity, *SD* standard deviation, *SV* stroke volume

*β coefficients were calculated using linear regression models adjusted age, smoking (ever), hypertension, and body mass index (BMI)

## Discussion

In this cross-sectional study, we found no differences in arch PWV or LV systolic function measures between middle-aged women with ischemic stroke, with or without migraine, nor with women without stroke or migraine. Although arch PWV was slightly elevated in the ischemic stroke with migraine group and minor reductions in SV, CO, and EF were observed in the ischemic stroke groups with and without migraine compared with the reference group, these differences were not statistically significant.

Previous studies have shown mixed results regarding aortic and arterial stiffness in young to middle-aged patients with migraine who are free of cardiovascular risk factors [10–12]. While one study reported a decrease in aortic stiffness in migraine without aura, others found no difference between migraine subtypes and migraine-free controls. However, to our knowledge, no prior studies have investigated the association between migraine—either alone or in patients with ischemic stroke—and LV systolic function measures. In addition, stroke etiologies differed somewhat between groups, with a higher proportion of undetermined stroke among women with migraine. This is in line with our previous CREW-MIST and WHISPER substudy, which also suggested a different etiologic profile in women with stroke and migraine, including more frequent undetermined stroke and less smoking compared with women with stroke alone [14]. As these stroke mechanisms are less likely to involve atherosclerosis, such etiologic heterogeneity may partly explain the absence of an association between migraine and arterial stiffness or LV systolic function observed in this study, and may have influenced the interpretation of macrovascular differences between groups [23]. 

Aortic stiffness can be assessed using various methods, including invasive aortic PWV, the gold standard but rarely used due to its invasiveness, and carotid-femoral PWV, the recommended noninvasive method for research [24]. In this study, MRI was chosen to measure arch PWV, as it provides precise measurement and allows simultaneous assessment of other cardiovascular parameters such as LV function. The added value of MRI lies in its ability to noninvasively evaluate localized stiffness in the aortic arch and accounts for true path length and regional elasticity, which is particularly relevant in understanding the relationship between aortic stiffness and ischemic stroke, where localized arterial stiffness may play a key role [21]. 

Our study has several limitations. First, the cross-sectional design precludes any inference about causality or temporal relationships between migraine, arch PWV, LV systolic function, and ischemic stroke. Longitudinal data are therefore required to assess temporal changes and clarify their potential causal role in stroke risk among women with migraine. In addition, stroke etiologies differed between groups, with more undetermined causes among women with migraine, which may limit the relevance of macrovascular markers for between-group comparisons. Because the number of patients with arterial dissection or patent foramen ovale was small (combined *n* = 6), we could not further evaluate these specific stroke mechanisms, although they may be particularly relevant in the context of migraine. Second, we included only women due to the higher prevalence of migraine in this population, which limits the generalizability of our findings to men. However, this focus is clinically more relevant, as the risk of ischemic stroke is higher in women, particularly in those with migraine with aura, whereas this association is less clear in men [3]. Third, the small sample size may have limited our ability to detect differences in cardiovascular measures between groups. The reference group included fewer participants than planned (*n* = 30 vs. the prespecified 38), potentially increasing the risk of a type II error. Although both stroke groups met the required sample size, the smaller reference group may have contributed to underpowering. However, while the original sample size calculation assumed a standard deviation of 3.1 m/s for aortic arch PWV, the observed variability was substantially lower (SD ≈ 1.5 m/s). Consequently, the statistical power to detect a clinically meaningful absolute difference of 2.0 m/s was higher than originally anticipated, despite the imbalance in group sizes; under these conditions, approximately 10 participants per group would have been sufficient to achieve 80% power. However, because the observed between-group differences in arch PWV were smaller than the prespecified clinically meaningful difference of 2.0 m/s, the study was mainly powered to detect relatively large effects and may have lacked power to detect smaller differences. Therefore, a type II error for modest between-group differences cannot be excluded. Given the exploratory nature of this study and the small sample size, analyses were interpreted cautiously and conducted as complete case analyses without adjustments for multiple testing [25]. Fourth, our findings may have been influenced by unmeasured confounders, including heart rate, medication use, and stroke severity or other stroke characteristics. These variables were not included because data on heart rate, medication use, NIHSS at presentation, and lesion volume were not available in the dataset. Therefore, residual confounding cannot be excluded. Subgroup analyses, including comparisons between migraine subtypes, were further restricted by the small sizes of the ischemic stroke groups. While including younger women (20–40 years) would have been of interest, the low occurrence of stroke in this age group and the advanced age of our cohort may have overshadowed potential differences detectable at a younger age. As a result, our findings are limited to women aged 40–60 years, further narrowing the generalizability of the study. Fifth, selection bias may have been introduced as this is a clinic-based rather than a population-based study, with different recruitment strategies for women with ischemic stroke and the reference group. Women with ischemic stroke, with or without migraine, were recruited through neurology clinics, whereas the reference group was recruited through advertisements by the Dutch Heart Foundation. These differences in recruitment may have resulted in a selected reference group that was not fully comparable to the stroke groups, which could have influenced the observed between-group differences. Sixth, because migraine characteristics were based on patient-reported information, there is potential for recall bias.

The strengths of this study include the use of advanced imaging techniques to assess macrovascular health in these specific populations for the first time, providing precise measurements of parameters, whereas most previous studies have focused on the microvascular system. We also used a standardized migraine diagnosis based on ICHD-III criteria and standardized measurements of arch PWV and LV systolic function from two cross-sectional studies. By focusing on middle-aged women, we targeted a population with an elevated stroke risk yet minimal influence from age-related cardiovascular risk factors and atherosclerosis, enabling a clearer assessment of macrovascular health.

## Conclusion

In this study, no association between migraine and macrovascular dysfunction, as assessed by arch PWV and LV systolic function measures, in middle-aged women with ischemic stroke was detected. Given the cross-sectional design and the limited sample size, these findings should be interpreted with caution and should not be taken as evidence for absence of an effect. Alternative pathways, such as microvascular dysfunction, may also contribute to the link between migraine and ischemic stroke, particularly in patients with low traditional cardiovascular risk scores.

## Supplementary Information

Supplementary Material 1.

Supplementary Material 2.

## Data Availability

The data supporting the findings of this study are available from the corresponding author upon reasonable request.

## Abbreviations

BMI: Body Mass Index
CI: Confidence Interval
CMR: Cardiovascular Magnetic Resonance
CO: Cardiac Output
CREW: MIST–Cardiovascular RiskprofilE in Women–MIcrovascular STatus
EF: Ejection Fraction
ICHD: III–International Classification of Headache Disorders, 3rd Edition
LUMC: Leiden University Medical Center
LV: Left Ventricular
MRI: Magnetic Resonance Imaging
PWV: Pulse Wave Velocity
SV: Stroke Volume
STROBE: Strengthening the Reporting of Observational Studies in Epidemiology
TOAST: Trial of ORG 10172 in Acute Stroke Treatment
WHISPER: WHIte matter lesions in young to middle–aged women with Stroke, PreEclampsia, and migRaine
