# Supplementary material for: Aortic stiffness and cardiac function in middle-aged women with ischemic stroke with and without migraine
Source: BMC Neurol. 2026 May 23;26:462. doi: 10.1186/s12883-026-04960-z (PMC13374289; doi:10.1186/s12883-026-04960-z)
Supplement: Supplementary file 1 — Supplementary Material 1. [file 12883_2026_4960_MOESM1_ESM.docx]

**Appendix**

CREW member list

The CREW consortium consists of (in alphabetical order):

Yolande Appelman^1^, Sara Baart^2,3^, Laura Benschop^2,3^, Eric Boersma^2^, Laura Brouwers^3,4^, Ricardo Budde^2^, Suzanne Cannegieter^5^, Veerle Dam^3,6^, Rene Eijkemans^6^, Bart Fauser^4^, Michel Ferrari^5^, Arie Franx^3^, Christianne de Groot^1^, Marlise Gunning^3,4^, Annemieke Hoek^7^, Erik Koffijberg^6,8^, Wendy Koster^2^, Mark Kruit^5^, Giske Lagerweij^3,6^, Nils Lambalk^1^, Joop Laven^2^, Katie Linstra^2,3,5^, Aad van der Lugt^2^, Angela Maas^9^, Antoinette Maassen van den Brink^2^, Cindy Meun^2,3^, Saskia Middeldorp^10^, Karel GM Moons^6^, Bas van Rijn^4^, Jeanine Roeters van Lennep^2^, Jolien Roos-Hesselink^2^, Luuk Scheres^3,10^, Yvonne T. van der Schouw^6^, Eric Steegers^2^, Regine Steegers^2^, Gisela Terwindt^5^, Birgitta Velthuis^3^, Marieke Wermer^5,7^, Bart Zick^2,5^, Gerbrand Zoet^3,4^

^1^Amsterdam UMC – location VUmc, Amsterdam, the Netherlands

^2^Erasmus MC University Medical Center, Rotterdam, the Netherlands

^3^Netherlands Heart Institute, Utrecht, the Netherlands

^4^University Medical Center Utrecht, Utrecht, the Netherlands

^5^Leiden University Medical Center, Leiden, the Netherlands

^6^Julius Center, Utrecht, University Medical Center, Utrecht, the Netherlands

^7^University Medical Center Groningen, Groningen, the Netherlands

^8^University of Twente, Enschede, the Netherlands

^9^Radboud University Medical Center, Nijmegen, the Netherlands

^10^Amsterdam UMC – location AMC, Amsterdam, the Netherlands
